# The impact of long-term ventilator-use on health-related quality of life and the mental health of children with neuromuscular diseases and their families: need for a revised perspective?

**DOI:** 10.1186/s12955-020-01467-0

**Published:** 2020-07-09

**Authors:** Jessika Johannsen, Lena Fuhrmann, Benjamin Grolle, Lydia Morgenstern, Silke Wiegand-Grefe, Jonas Denecke

**Affiliations:** 1 Department of Pediatrics, University Medical Center Hamburg-Eppendorf, Hamburg, Martinistr, 52 20246 Hamburg, Germany; 2 Children’s Hospital Altona, Hamburg, Germany; 3 Department of child and adolescent psychiatry, psychosomatics and psychotherapy, University Medical Center Hamburg-Eppendorf, Hamburg, Germany

## Abstract

**Background:**

Life extension by medical interventions and health-related quality of life (HRQOL) are sometimes conflicting aspects of medical care. Long-term ventilation in children with neuromuscular disease is a well-established life-extending procedure and often at the center of this conflict. HRQOL and the mental health of affected children and their families become even more important in respect to emerging therapies in neuromuscular diseases with longer life-expectancy of treated patients and considerable costs of medical treatment.

**Methods:**

We performed a questionnaire survey in a total of forty-three families of children with neuromuscular disease treated in the University Medical Center Hamburg-Eppendorf and the Children’s Hospital Altona. We evaluated self- and proxy-reported HRQOL and mental health outcomes of affected children and their parents using validated and age-appropriate instruments.

**Results:**

Compared to normative data, children with neuromuscular diseases and their families experienced a lower HRQOL and mental health. However, there was no additional negative influence on the overall HRQOL by ventilator use.

**Conclusions:**

As ventilator use was not responsible for the reduction of HRQOL and mental health our data contributes an important aspect to the discussion about life-prolonging procedures, in particular mechanical ventilation, in severly disabled patients.

## Background

Neuromuscular disorders are mostly life-limiting diseases using mechanical ventilation at any stage as a tool of palliative care extending life expectancy of the patients [1–3]. In recent years a growing number of new therapies in the field of neuromuscular diseases have emerged modifying the disease’s course and enhancing the life-expectancy of affected children [4–6]. In this context two aspects of mechanical ventilation in those patients become more important: 1. Extending patient’s life expectancy with the option that new therapeutics are available in the future and 2. Focusing on the quality of life considering that new therapies might extend life expectancy but still do not reverse the course of the disease to a significant extent. One example in this discussion is the initiation of mechanical ventilation in patients with spinal muscular atrophy type 1 in the face of recently approved disease-modifying treatments such as nusinersen and/or Onasemnogene abeparvovec [4–7]. In everyday clinical practice, healthcare economy as well as the knowledge of patients and health care professionals are additional factors in the decision for or against ventilation in patients with neuromuscular disorders at any stage of the disease. How health care professionals’ and caregivers’ assess a patients’ quality of life is crucial for medical counseling. However, HRQOL of technology-dependent and/or physically disabled children were reported to be significantly underestimated by caregivers [8, 9].

The care for a technology-dependent child leads to a significant burden on the caregivers and long-term dependence on medical equipment, which creates a considerable impediment of social life for the affected child and their families [10–13]. Moreover, an increased risk of externalizing and internalizing behavioural disorders, as well as developmental disorders and mental comorbidities were found in a longitudinal data analysis in families with technology-dependent children [14]. Additionally, parents of chronically ill children exhibit mental health problems and comorbidities due to the condition of their child, mostly in the spectrum of depression and anxiety disorders. The percentage of mothers with clinically significant anxiety and depression lies above 30 and 23% respectively [15]. Thus, the application of life-extending procedures, in particular ventilator use, must be embedded in the medical but also in the psychosocial context.

Considering this, the comparison of quality of life and mental health of non-ventilated and ventilated children with neuromuscular disease and their parents could be essential to the decision-making process of applying life-extending procedures. The purpose of our study was to investigate health-related quality of life and mental health outcomes of affected children and their families enclosing the aspect of ventilation. In total, 43 families of children with neuromuscular disease treated in the University medical center Hamburg-Eppendorf and the Children’s Hospital Altona were evaluated with standardized questionnaires and compared to a cohort of healthy children and children with other chronic diseases, respectively. In view of previous reports on health-related quality of life in ventilated patients [10, 12, 14, 16] we propose that long-term ventilation is one factor that impairs quality of life of affected children and their families due to an increase in disease burden.

## Methods

### Participants

Children and their parents were recruited during regularly scheduled clinic visits at the Department of Pediatrics, section neuropediatrics of the University Medical Center Hamburg-Eppendorf and the Pediatric Pulmonary Department of the Children’s Hospital Altona, Hamburg. The following 2 patient groups were included: (1) children unter the age of 21 years with neuromuscular disease with invasive and/or non-invasive ventilation, (2) children under the age of 21 years with neuromuscular disease without invasive and/or non-invasive ventilation.

Children and their parents with limited german-language comprehension or those who were unable to give informed consent were excluded. Families with children with cognitive impairment and/or severe additional diseases except the neuromuscular disease (for example epilepsy) were also excluded.

The following demographic information was collected by self-reported questionnaires and electronic health records: age of children and parents, gender of children, occupational status of parents, highest level of education of parents, family income, type of neuromuscular disorder, type and duration of mechanical ventilation.

Written, informed consent from the parents or guardians and assent from children who were able to read and understand the questionnaires were obtained before enrollment. Questionnaires were delivered to the families during the clinic visit or sent to the families’ home and were completed at home. The study was approved by the local ethics committee (PV4361).

### Measurement instruments

The primary study outcome was the mental health and health-related quality of life (HRQOL) of children and their parents. Mothers and fathers were interviewed separately.

In children, HRQOL was measured by the DISABKIDS-37 and the KIDSCREEN-27 questionnaires. The DISABKIDS-37 was developed as part of a set of instruments to assess self-reported HRQOL in European children aged 8 to 16 years with chronic health conditions and consists, in its long version (DCGM-37), of 37 likert-scaled items divided into the following scales: Independence, Emotion, Social inclusion, Social exclusion, Physical Limitation, and Treatment. In addition, this instrument provides a total score which represents an overall assessment of HRQOL. We used the questionnaire in the self-reported (child version) and the proxy version in german language [17]. The DCGM-37 showed acceptable to good internal consistencies (α = .70 to .87) and good to excellent test-retest reliabilities (ICC = .71 to .83) in a sample of children and adolescents with chronic health conditions [18] as well as acceptable internal consistencies in a pediatric sample undergoing cancer treatment, reflected by Cronbach’s alpha above .70 on all HRQOL subcategories [19].

In contrast, the KIDSCREEN questionnaire was developed to evaluate HRQOL of healthy children aged 8 to 18 years. The KIDSCREEN-27 short version covers five dimensions of HRQOL: Physical Well-Being, Psychological Well-Being, Autonomy & Parents, Peers & Social Support and School Environment. Again, we used the self-reported and the proxy version in german translation [20].

The children’s mental health was evaluated using the Strength and Difficulties questionnaire (SDQ, german translation). The SDQ is a behavioural screening questionnaire inquiring about 25 attributes and is available in the self-reported version for 11- to 16-year olds and the proxy version for parents with children between 4 and 16 years. The 25 items are divided between five scales of five items each, generating scores for Conduct Problems, Inattention/Hyperactivity, Emotional Symptoms, Peer Problems, and Prosocial Behavior. All scales except for the last are summed up to generate a Total Difficulties score [21]. Normative data of the German Health Interview and Exanimation Survey for children and adolescents (KiGGS) of the Robert Koch Institute were used for comparison [22].

The parental HRQOL was measured by the Ulmer Lebensqualitätsinventar für Eltern chronisch erkrankter Kinder (ULQIE). The ULQIE consists of 29 likert-scaled items containing five primary scales which cover five dimensions and a total score. The five primary scales are physical and daily functioning, satisfaction with the situation in the family, emotional distress, self-development, and well-being. Answers are given with regard to the preceding seven days on five-point rating scales ranging from 0 to 4 (‘never’ to ‘always’). Higher scores indicate better quality of life. The test was developed for measuring parental quality of life in family-centered intervention programs and was validated in a group of parents with children suffering from oncologic disease, diabetes mellitus and epilepsy [23].

Mental health of parents was determined by the Brief Symptom Inventory (BSI). It evaluates psychological distress using a 53-item self-reported scale covering nine symptom dimensions: Somatization, Obsession-Compulsion, Interpersonal Sensitivity, Depression, Anxiety, Hostility, Phobic anxiety, Paranoid ideation and Psychoticism. For analyses, the three global indices of distress Global Severity Index (GSI), Positive Symptom Distress Index (PSDI), and Positive Symptom Total (PST) are calculated. The global indices measure current or past level of symptomatology, intensity of symptoms, and number of reported symptoms, respectively [24, 25].

### Statistical analysis

The software IBM SSPS Statistics, version 22 was used to conduct all analyses. Missing values were filled up with the multiple imputation using the Markov Chain Monte Carlo (MCM) method. All analyses were performed for three different groups, consisting of, all families, (*n* = 43), families with ventilated patients (*n* = 18) and families with non-ventilated patients (*n* = 25) as well as separately for children (self-assessment and assessment by proxy) and their parents. For each variable, the violations of test assumptions, such as normality and homogeneity of variance were checked. T-Test for independent samples was used for comparison of averages between groups. The significance level was set at 0.05.

## Results

### Participants

Out of seventy-seven contacted families forty-three families returned the questionnaires. Eighteen (41.9%) families with ventilated children and 25 (48.1%) families with non-ventilated children. Demographic data, type of neuromuscular disease and ventilation status of the patients are shown in Table 1. Social standing of the families was comparable between groups. Ventilation treatment was initiated at the mean age of 4.42 years (SD: 5.44). In the ventilated group on average, children were ventilated for 13.21 h daily (SD: 6.53) and 4 of 18 (22.2%) children needed ventilation 24 h a day. Five (27.8%) children were ventilated since birth. In 13 (72.2%) children, ventilation had to be started because of disease progression at the mean age of 4.4 years (SD: 5.44). Six (33.3%) children were invasively ventilated via tracheostoma and 11 (61.1%) children were on non-invasive ventilation. In one child, information about the ventilation type was missing.

**Table 1:** Demographic characteristics, type of neuromuscular disease, ventilation status and response rates of patients

	children		
	total	ventilated	non-ventilated
**Number of individuals**	43	18	25
**Mean age in years (range)**	10.58 (1–21)	12.44 (1–21)	9.24 (2–17)
**Ventilator-use, n (%)**			
**Full time use**		4 (22.2)	
**nocturnal use**		8 (44.4)	
**nocturnal and intermittent daily use**		3 (16.7)	
**intermittent as required**		2 (11.1)	
**missing data**		1 (5.6)	
**Type of ventilation technology, n (%)**			
**non-invasive**		11 (61.1)	
**invasive**		6 (33.3)	
**missing data**		1 (5.6)	
**muscular dystrophy Duchenne, n (%)**	19 (44.2)	2 (11.1)	17 (68)
**other muscular dystrophy, n (%)**	8 (18.6)	1 (5.6)	7 (28)
**spinal muscular atrophy, n (%)**	6 (13.9)	6 (33.3)	0 (0)
**congenital myopathy, n (%)**	10 (23.3)	9 (50)	1 (4)
**Completing the questionnaire (HRQOL), n**			
**KIDSCREEN proxy version**	18	6	12
**KIDSCREEN self-reported**	21	11	10
**KIDSCREEN missing data**	4	1	3
**DISABKIDS proxy version**	18	6	12
**DISABKIDS self-reported**	21	11	10
**DISABKIDS missing data**	4	1	3
**Completing the questionnaire (mental health), n**			
**SDQ proxy version**	18	6	12
**SDQ self-reported**	21	11	10
**SDQ missing data**	4	1	3

Forty-two (97.67%) mothers (m_t_) completed the questionnaire regarding parents’ HRQOL and mental health compared to twenty-three (53.49%) fathers (f_t_). All mothers of ventilated children (m_v_, *n* = 18) and 24 (96%) mothers of non-ventilated children (m_nv_) answered the questionnaires. The response rate was lower in fathers with 7 (38.9%) fathers of ventilated children (f_v_) and 16 (64%) fathers of non-ventilated children (f_nv_) completing the questionnaires. For the proxy measures, questionnaires were completed in 77,8% by the mothers, in 11,1% by the father and in 11,1% data showing which parent answered the questionnaire were missing. Response rates of patients are summarised in Table 1.

### HRQOL of affected children

**Fig. 1:**
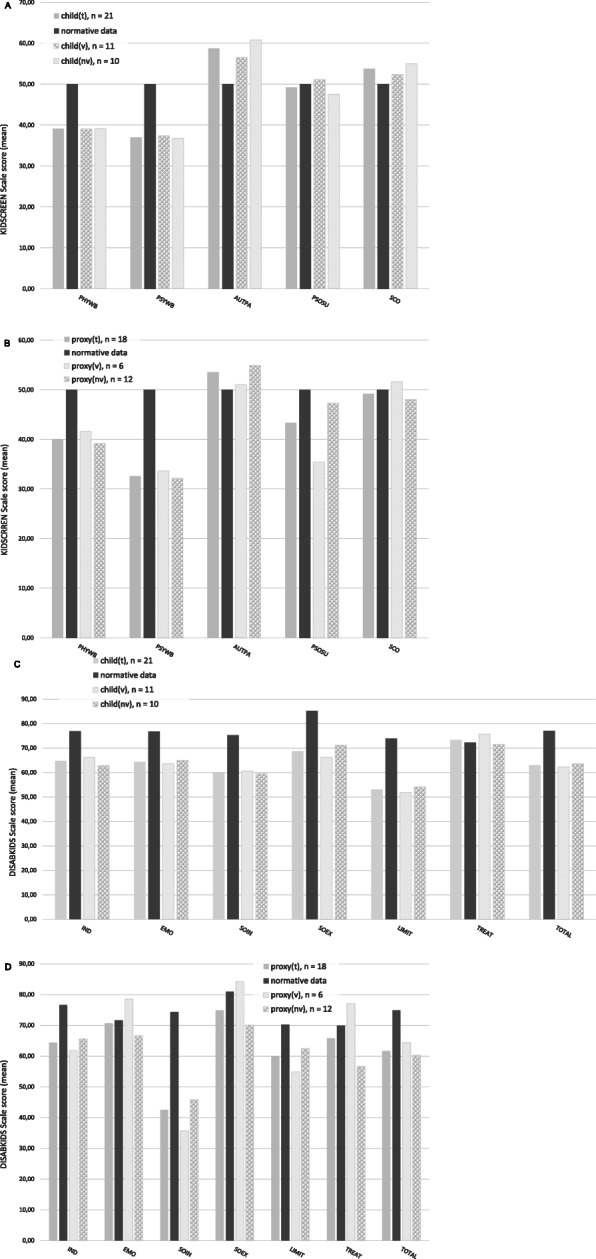
Health-related quality of life reported by the children (self-reported) and by proxy (parents) respectively, measured with the German version of the KIDSCREEN-27 and the DISABKIDS-37. **a** self-reported HRQOL, measured by the KIDSCREEN-27; all children (child_t_, *n* = 21), ventilated children (child_v_, *n* = 11), non-ventilated children (child_nv_, *n* = 10); **b** HRQOL of the children reported by their parents, measured by the KIDSCREEN-27: all children (proxy_t_, *n* = 18), ventilated children (proxy_v_, *n* = 6), non-ventilated children (proxy_nv_, *n* = 12); **c** self-reported HRQOL, measured by the DISABKIDS-37; all children (child_t_, n = 21), ventilated children (child_v_, n = 11), non-ventilated children (child_nv_, n = 10); **d** HRQOL of children reported by their parents, measured by the DISABKIDS-37; all children (proxy_t_, *n* = 18), ventilated children (proxy_v_, n = 6), non-ventilated children (proxy_nv_, n = 12). Abbreviations: KIDSCREEN-27: PHYWB, Physical Well-Being; PSYWB, Psychological Well-Being; AUTPA, Autonomy and Parents; PSOSU, Peers and Social Support; SCO, School Environment. DISABKIDS-37: IND, Independence; EMO, Emotion; SOIN, Social Inclusion; SOEX, Social Exclusion; LIMIT, Limitation, TREAT, Treatment; TOTAL total quality of life

Measured by the KIDSCREEN-27, children with neuromuscular diseases (child_t_, *n* = 21) reported lower scores in Physical (M = 39.1, SD = 6.22; *p* = .000) and Psychological Well-Being (M = 37.05, SD = 3.24; p = .000) than in the norm group of healthy children (M = 50, SD = 10). In children with neuromuscular diseases, scores in Autonomy & Parents (M = 58.76, SD = 10.28; *p* = .001) and in School Environment (M = 53.74, SD = 7.88; *p* = .042) were higher than in healthy children. Similar results with lower scores of Physical and Psychological Well-Being and a higher score in Autonomy & Parents were found in both sub-groups of ventilated (child_v,_*n* = 11, Physical Well-Being (M = 39.15, SD = 7.52; *p* = .001), Psychological Well-Being (M = 36.76, SD = 3.14; *p* = .000), Autonomy & Parents (M = 60.8, SD = 11.73; *p* = .012)) and non-ventilated children (child_nv_, *n* = 10, Physical Well-Being (M = 39.05, SD = 4.81; p = .000), Psychological Well-Being (M = 37.37, SD = 3.5; p = .000), Autonomy & Parents (M = 56.52, SD = 8.46; *p* = .038)) compared to healthy children (Fig. 1a).

Consistently, parents of all children with neuromuscular diseases (proxy_t_, *n* = 18) reported lower scores in Physical (M = 39.97, SD = 8.09; *p* = .000) and Psychological Well-being (M = 32.63, SD = 3.75; *p* = .000) of their children compared to healthy children (Physical Well-being (M = 49.98, SD = 10.01), Psychological Well-being (M = 49.99, SD = 10)). In sub-groups, lower scores of Psychological Well-being (M = 33.62, SD = 3.3; *p* = .000) in ventilated children (proxy_v,_*n* = 6) and lower scores of Physical Well-being (M = 39.16, SD = 6.13; p = .000) and Psychological Well-being (M = 32.13, SD = 4.0; p = .000) in non-ventilated children (proxy_nv_, *n* = 12) compared to norm data of healthy children (Physical Well-being (M = 49.98, SD = 10.01), Psychological Well-being (M = 49.99, SD = 10) were confirmed by the parents. Additionally, parents stated a lower score in Peers & Social Support (M = 43.33, SD = 11.60) (*p* = .026) of their children with neuromuscular diseases than parents of healthy children (M = 50, SD = 10). This was even lower (*p* = .035) in the sub-group of ventilated children (M = 35.38, SD = 15.33) compared to non-ventilated children (M = 47.30, SD = 7.02) (Fig. 1b).

Self-reported quality of life measured by the DISABKIDS-37 was significantly lower in total (Global score M = 62.94, SD = 17.39; *p* = .001) and in all sub-scales except Treatment in children with neuromuscular disease (child_t_, *n* = 21) (Independence (M = 64.68, SD = 19.21; *p* = .009), Emotion (M = 64.29, SD = 20.86; *p* = .013), Social inclusion (M = 60.12, SD = 20.69; *p* = .003), Social exclusion (M = 68.85, SD = 20.57; p = .001), Physical Limitation (M = 52.98, SD = 17.93; *p* = .000)) compared to norm data of children with other chronic diseases (Global score (M = 76.99, SD = 14.22), Independence (M = 76.9, SD = 18.34), Emotion (M = 76.72, SD = 20.56), Social inclusion (M = 75.25, SD = 17.81), Social exclusion (M = 85.1, SD = 15.56), Physical Limitation (M = 73.85, SD = 18.23)). In comparison with chronically ill children of the norm data group, in ventilated children (child_v_, *n* = 11) overall score (M = 62.32, SD = 19.04; *p* = .029) and scores in Social inclusion (M = 60.61, SD = 20.19; *p* = .037), Social exclusion (M = 66.29, SD = 19.23; *p* = .009) and Physical Limitation (M = 51.98, SD = 18.2; *p* = .003) were lower, similar results were found in non-ventilated children (child_nv_, *n* = 10) in overall score (M = 63.63, SD = 16.39; *p* = .030) and in scores of Emotion (M = 65.00, SD = 15.32; *p* = .039) and Limitation (M = 54.17, SD = 18.35; *p* = .008). Scores were not significantly different between ventilated and non-ventilated children (Fig. 1c).

According to their parents, children with neuromuscular disease (proxy_t_, *n* = 18) are significantly impaired in total (M = 61.66, SD = 11.76; *p* = .000) and in the scales Independence (M = 64.35, SD = 14.52; *p* = .002), Social Inclusion (M = 42.44, SD = 14.2; p = .000) and Physical Limitation (M = 59.95, SD = 15.99; *p* = .015) compared to children with other chronic diseases of the norm data group (total (M = 74.90, SD = 14.55), Independence (M = 76.7, SD = 17.25), Social Inclusion (M = 74.28, SD = 17.65), Physical Limitation (M = 70.19, SD = 18.32)). Lower scores of Social inclusion (M = 35.67, SD = 16.42; *p* = .002) and Physical limitation (M = 54.86, SD = 11; *p* = .019) were also reported by the parents in the sub-group of ventilated children (proxy_v_, *n* = 6) compared to the norm group of children with chronic diseases. In non-ventilated children (proxy_nv_, *n* = 12) not only the overall score (M = 60.3, SD = 11.97; *p* = .001) and the scores in Independence (M = 65.63, SD = 13.89; p = .019) and Social inclusion (M = 45.83, SD = 12.31; *p* = .000) were lower than in children with chronic disease but also the score in Treatment (M = 56.67, SD = 21.37 vs. M = 69.9, SD = 22.15; p = .001). There was no significant difference between non-ventilated and ventilated children (Fig. 1d).

### Mental health of affected children

**Fig. 2:**
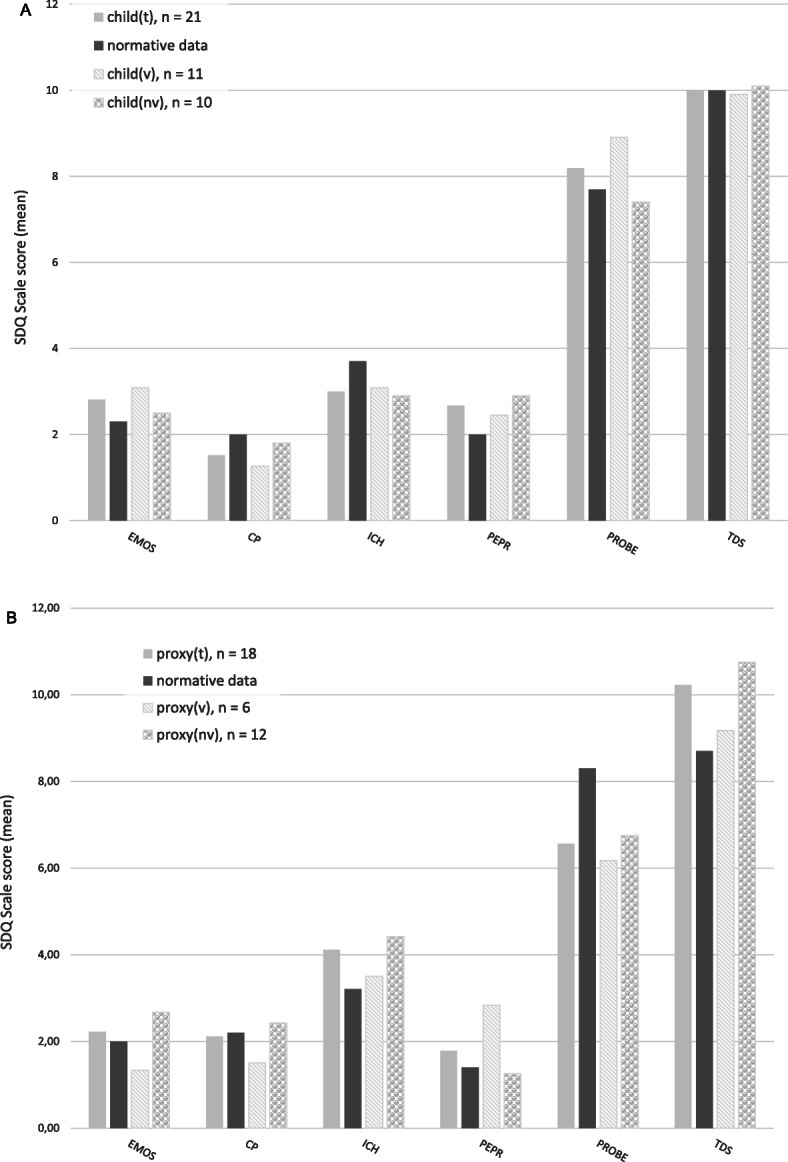
Mental health of children, measured by the German version of the SDQ. **a** reported by the children (self-reported), all children (child_t_, n = 21), ventilated children (child_v_, n = 11),.non-ventilated children (child_nv_, n = 10); **b** reported by proxy (parents), all children (proxy_t_, n = 18), ventilated children (proxy_v_, n = 6), non-ventilated children (proxy_nv_, n = 12). Abbreviations: CP, Conduct Problems; ICH, Inattention/Hyperactivity; EMOS, Emotional Symptoms; PEPR, Peer Problems; PROBE, Prosocial Behavior; TDS Total Difficulties Score

Using the SDQ, self-reported mental health scores in all children with neuromuscular disease (child_t_, *n* = 21) and in the sub-group of non-ventilated children (child_nv_, *n* = 10) were similar to healthy children. Though, ventilated children (child_v_, *n* = 11) reported lower scores measuring conduct problems (M = 1.27, SD = 0.79; *p* = .024) than healthy children (M = 1.4, SD = 0.79) and significantly higher scores scaling prosocial behaviour (M = 8.91, SD = 1.22; *p* = .008) compared to healthy children (M = 7.70, SD = 1.70; p = .008) and to non-ventilated children (M = 7.40, SD = 1.71; *p* = .03) ((Fig. 2a).

Parents estimated the prosocial behaviour of their children (proxy_t_, *n* = 18, M = 6.56, SD = 1.79, *p* = .001; proxy_v_, *n* = 6, M = 6.1, SD = 1.49, *p* = .015; proxy_nv_, *n* = 12, M = 6.75, SD = 1.96, *p* = .019) to be significantly lower compared to the norm group of children (M = 8.3, SD = 0.02). More problems with peers were reported by parents in their ventilated children (M = 2.83, SD = 1.47) than in non-ventilated children (M = 1.25, SD = 1.36) (*p* = .037) (Fig. 2b).

### HRQOL of mothers and fathers

**Fig. 3:**
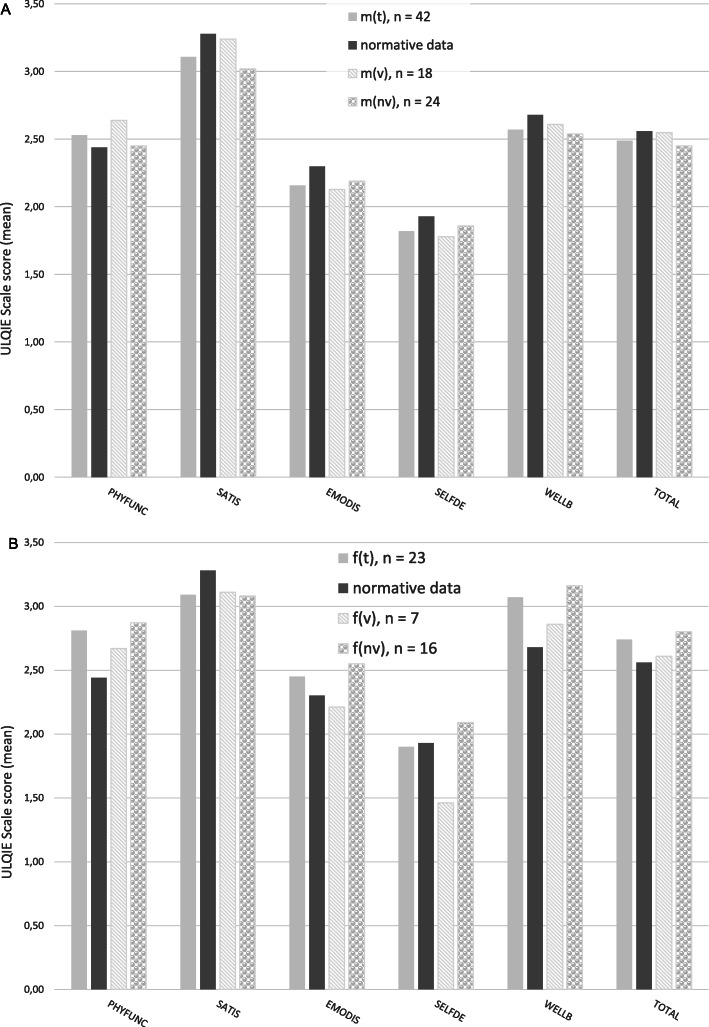
Health-related quality of life of parents, measured by the ULQIE. **a** HRQOL reported by mothers, all mothers (m_t_, *n* = 42), mothers of ventilated children (m_v_, n = 18), mothers of non-ventilated children (m_nv_, *n* = 24); **b** HRQOL reported by fathers, all fathers (f_t_, *n* = 23), fathers of ventilated children (f_v_, *n* = 7), fathers of non-ventilated children (f_nv_, *n* = 16). Abbreviations: PHYFUNC, physical and daily functioning; SATIS, satisfaction with the situation in the family; EMODIS, emotional distress; SELFDE, self-development; WELLB, well-being; TOTAL, total score

In the group of all mothers (m_t_, *n* = 42) irrespective if the child is ventilated (m_v_, *n* = 18) or not (m_nv_, *n* = 24) no differences were found in any of the 5 dimensions of the ULQIE compared to parents with chronically ill children of the norm group. Mothers with ventilated children and mothers with non-ventilated children showed no differences in HRQOL as well (Fig. 3a).

In the group of fathers (f_t_, *n* = 23), higher scores were reached in physical and daily functioning (M = 2.81, SD = 0.74; *p* = .026) and well-being (M = 3.07, SD = 0.67; *p* = .011) compared to normative data of parents with chronically ill children (physical and daily functioning (M = 2.44, SD = 0.72), well-being (M = 2.68, SD = 0.69)). Compared to parents with chronically ill children, no differences in HRQOL were found in fathers with ventilated children (f_v_, *n* = 7), but fathers with non-ventilated children (f_nv_, *n* = 16) showed higher scores in physical and daily functioning (M = 2.87, SD = 0.6; *p* = .012), well-being (M = 3.16, SD = 0.5; *p* = .002) and in the total scale (M = 2.8, SD = 0.41 vs. M = 2.56, SD = 0.53; *p* = .03). Self-development was significantly higher scaled in the group of fathers with non-ventilated children (M = 2.09, SD = 0.55) compared to fathers with ventilated children (M = 1.46, SD = 0.71) (*p* = .032) (Fig. 3b).

### Mental health of mothers and fathers

**Fig. 4:**
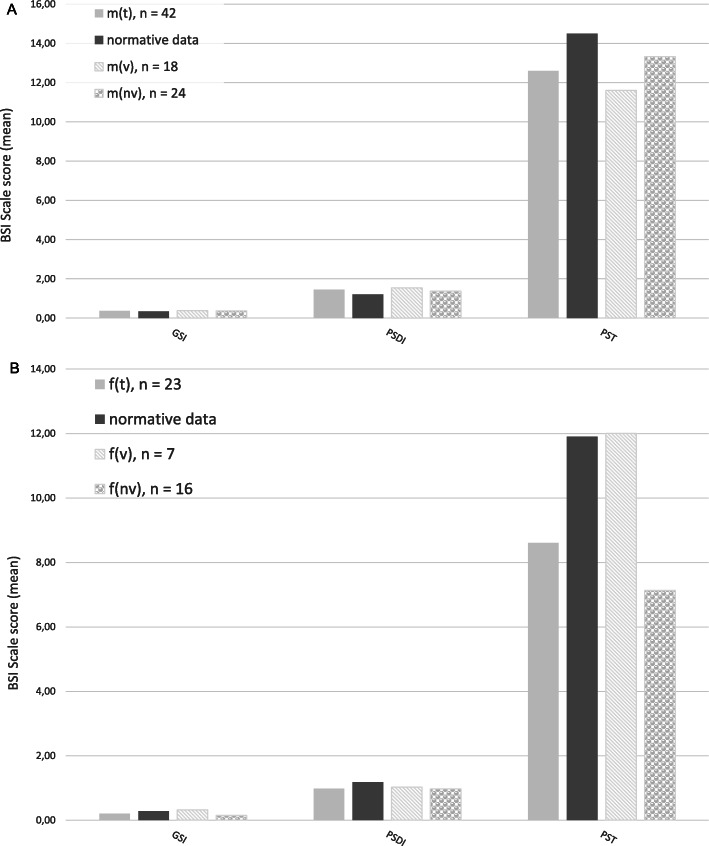
Mental health of parents, measured by the BSI. **a** reported by mothers, all mothers (m_t_, n = 42), mothers of ventilated children (m_v_, n = 18), mothers of non-ventilated children (m_nv_, n = 24); **b** reported by fathers, all fathers (f_t_, n = 23), fathers of ventilated children (f_v_, n = 7), fathers of non-ventilated children (f_nv_, n = 16). Abbreviations: GSI, Global Severity Index; PSDI, Positive Symptom Distress Index; PST, Positive Symptom Total

In comparison with normative data, mothers in our group (m_t_, *n* = 42) showed lower scores in the scale Psychoticism (M = 0.12, SD = 0.21 vs. M = 0.19, SD = 0.27; *p* = .049). Moreover, mothers with ventilated children (m_v_, *n* = 18) expressed lower scores in the scales Interpersonal Sensitivity (M = 0.32, SD = 0.33; *p* = 0.42) and Psychoticism (M = 0.09, M = 0.02; p = .04) and mothers with non-ventilated children (m_nv_, *n* = 24) had lower scores in the dimension Phobic anxiety (M = 0.07, SD = 0.13; *p* = .002) compared to normative data (Interpersonal sensitivity M = 0.49, SD = 0.45; Psychoticism M = 0.19, SD = 0.27; Phobic anxiety M = 0.16, SD = 0.25) (Fig. 4a).

Fathers (f_t_, *n* = 23) of children with neuromuscular disease reported lower mental stress with lower scores in PSDI (M = 0.98, SD = 0.44; *p* = .043), Interpersonal Sensitivity (M = 0.17, SD = 0.27; *p* = .004), Anxiety (M = 0.13, SD = 0.15; *p* = .000), Hostility (M = 0.20, SD = 0.18; *p* = .026), Paranoid Ideation (M = 0.19, SD = 0.22; *p* = .007), compared to normative data (PSDI M = 1.18, SD = 0.33, Interpersonal Sensitivity M = 0.35, SD = 0.40, Anxiety M = 0.29, SD = 0.31, Hostility M = 0.29, SD = 0.35, Paranoid Ideation M = 0.33, SD = 0.40). In the sub-groups, fathers with non-ventilated children (f_nv_, *n* = 16) revealed lower mental stress in almost all dimension and in the global indices GSI and PST compared to normative data (Obsession Compulsion M = 0.26, SD = 0.23 vs. M = 0.50, SD = 0.46; *p* = .001; Interpersonal Sensitivity M = 0.13, SD = 0.18 vs. M = 0.35, SD = 0.40; *p* = .000; Anxiety M = 0.11, SD = 0.12 vs. M = 0.29, SD = 0.31; p = .000; Hostility M = 0.20, SD = 0.15 vs. M = 0.29, SD = 0.35; *p* = .026; Phobic anxiety M = 0.05, SD = 0.12 vs. M = 0.14, SD = 0.23; *p* = .007; Paranoid Ideation M = 0.16, SD = 0.21 vs. M = 0.33, SD = 0.40; *p* = .006; Psychoticism M = 0.06, SD = 0.12 vs. M = 0.19, SD = 0.28; p = .001; GSI M = 0.15, SD = 0.1 vs. M = 0.28, SD = 0.23; p = .000; PST M = 7.13, SD = 4.91 vs. M = 11.90, SD = 8.10; p = .001). No differences in mental health between fathers with ventilated children (f_v_*n* = 7) and with non-ventilated children f_nv_ (*n* = 16) were found (Fig. 4b).

## Discussion

In face of the growing number of disease-modifying therapies in the field of neuromuscular disorders the quality of life of patients and their families is of central importance for a decision on life-prolonging procedures, such as mechanical ventilation, and the initiation of these therapies. However, previous studies have stated, that HRQOL of technology-dependent and/or physically disabled children might be significantly underestimated by the caregivers [8, 9].

Therefore, we evaluated the quality of life and mental health of families with ventilated and non-ventilated children with neuromuscular diseases. As reported by others [12, 13, 26–29], children with neuromuscular diseases and their parents reported a reduced child’s overall quality of life especially regarding the Physical and Psychological Well-Being and Social integration and also in some aspects of their mental health compared to healthy children. Moreover, quality of life of our patients was even lower than in the group of children with other chronic diseases. Although technical equipment, for example portable medical devices and long-lasting batteries, has improved over time, our results might reflect the impact of neuromuscular disorders on managing daily life and the proper integration of physically disabled children in their social environment. Surprisingly and in contrast to our hypothesis, these results were irrespective of the need of mechanical ventilation and confirm that ventilator use per se did not negatively influence health-related quality of life and mental health in our patients. This seems counterintuitive but is consistent with previous reports about the improvement of quality of life after initiation of mechanical ventilation in patients with neuromuscular disease [30, 31]. It also supports our experience in clinical practice with positive effects on physical performance due to mechanical ventilation, mainly in children using non-invasive ventilation in later stages of the disease such as Duchenne muscular dystrophy. Seear et al. (2016) also reported only mild to moderate adverse effects of mechanical ventilation on the quality of life in the majority of home-ventilated children with heterogeneous underlying diseases [13]. Indeed, our results might be influenced by the higher proportion of children with non-invasive ventilation (61.1%) as this might have a lower impact on daily life compared to invasive ventilation. However, some authors associated ventilator use in children with significant lower scores in most aspects of health-related quality of life [12, 16, 26]. These studies included a broader spectrum of underlying medical conditions impairing comparability with our data as the underlying disease itself might contribute to the impairment of quality of life. Mah et al. showed poorer physical and psychosocial HRQOL in Canadian ventilated children compared to non-ventilated children with neuromuscular diseases. Children with home ventilation counted for only 17% of the study population. Authors interpreted their results as a consequence of disease severity and reduced involvement in social activities of ventilated children [32]. In our population, reduced contact to peers in ventilated children compared to non-ventilated children was reported only by the parents but not by the children themselves. Similar results with fewer relationships with friends were also reported by parents for their home-ventilated children in the study of Noyes et al. (2007) [16]. This might reflect differences in child self-report and parent proxy measures as indicated by Bray (2017) [9]. HRQOL and mental health outcomes of parents in our population were only to some extent decreased. These results were again not dependent on the use of mechanical ventilation in their children. In fact, in many domains the parents and here especially the fathers scored higher than the normative groups. Though, we realized high scores in the Positive Symptom Distress Index that might point towards the tendency to give positive self-descriptions as decribed by Paulhus (2002) [33]. Another explanation might be offered by the Theory of social comparison established by Festinger (1954) [34]. The theory states, that individuals assess their own situation and abilities in relation to others. Assuming that families with chronically ill children are more often in hospital and in contact with families in similar circumstances, their own distress might be modified. Moreover, influencing factors as social support, family dynamics and relations might influence our results but have not been evaluated separately.

Although our study has some limitations due to the small sample size and the heterogenous group of patients regarding the underlying neuromuscular diseases and the kind and duration of mechanical ventilation it should be considered as assisting data, that might influence the perspective on ventilator-use as a life-extending procedure in children with neuromuscular diseases. As the debate about the use of long-term ventilation in severly affected children with neuromuscular disease is still polarizing and often colored by individual experience the results in our patients emphasizes, that ventilator use per se is not the limiting factor for the HRQOL and mental health of affected children and their families.

## Conclusions

Quality of life of patients and families must be a central decision criterium for medical interventions especially in palliative care situations. Therefore, our data contributes an important aspect in the controversial discussion about establishing life-prolonging procedures since ventilator use was not responsible for the reduction of HRQOL and mental health in our population of children with neuromuscular diseases and their families.

## Data Availability

The datasets used and/or analysed during the current study are available from the corresponding author on reasonable request.

## Abbreviations

HRQOL: Health-related quality of life
SMA: Spinal muscular atrophy
SDQ: Strength and Difficulties questionnaire
ULQIE: Ulmer Lebensqualitätsinventar für Eltern chronisch erkrankter Kinder
GSI: Global Severity Index
PSDI: Positive Symptom Distress Index
PST: Positive Symptom Total
DMD: Duchenne muscular dystrophy
PHYWB: Physical Well-Being
PSYWB: Psychological Well-Being
AUTPA: Autonomy and Parents
PSOSU: Peers and Social Support
SCO: School Environment
IND: Independence
EMO: Emotion
SOIN: Social inclusion
SOEX: Social exclusion
LIMIT: Limitation
TREAT: Treatment
CP: Conduct Problems
ICH: Inattention/Hyperactivity
EMOS: Emotional Symptoms
PEPR: Peer Problems
PROBE: Prosocial Behavior
TDS: Total Difficulties Score
PHYFUNC: Physical and daily functioning
SATIS: Satisfaction with the situation in the family
EMODIS: Emotional distress
SELFDE: Self-development
WELLB: Well-being
TOTAL: Total score
